# Epigenetic landscape of germline specific genes in the sporophyte cells of *Arabidopsis thaliana*

**DOI:** 10.3389/fpls.2015.00328

**Published:** 2015-05-13

**Authors:** Chol Hee Jung, Martin O'Brien, Mohan B. Singh, Prem L. Bhalla

**Affiliations:** ^1^Plant Molecular Biology and Biotechnology Laboratory, Melbourne School of Land and Environment, The University of MelbourneParkville, VIC, Australia; ^2^VLSCI Life Sciences Computation Centre, The University of MelbourneParkville, VIC, Australia

**Keywords:** epigenetic, germline, gene repression, histones, gametophyte, sporophyte

## Abstract

In plants, the germline lineages arise in later stages of life cycle as opposed to animals where both male and female germlines are set aside early in development. This developmental divergence is associated with germline specific or preferential expression of a subset of genes that are normally repressed for the rest of plant life cycle. The gene regulatory mechanisms involved in such long-term suppression and short-term activation in plant germline remain vague. Thus, we explored the nature of epigenetic marks that are likely associated with long-term gene repression in the non-germline cells. We accessed available *Arabidopsis* genome-wide DNA methylation and histone modification data and queried it for epigenetic marks associated with germline genes: genes preferentially expressed in sperm cells, egg cells, synergid cells, central cells, antipodal cells or embryo sac or genes that are with enriched expression in two or more of female germline tissues. The vast majority of germline genes are associated with repression-related epigenetic histone modifications in one or more non-germline tissues, among which H3K9me2 and H3K27me3 are the most widespread repression-related marks. Interestingly, we show here that the repressive epigenetic mechanisms differ between male and female germline genes. We also highlight the diverse states of epigenetic marks in different non-germline tissues. Some germline genes also have activation-related marks in non-germline tissues, and the proportion of such genes is higher for female germline genes. Germline genes include 30 transposable element (TE) loci, to which a large number of 24-nt long small interfering RNAs were mapped, suggesting that these small RNAs take a role in suppressing them in non-germline tissues. The data presented here suggest that the majority of *Arabidopsis* gamete-preferentially/-enriched genes bear repressive epigenetic modifications or regulated by small RNAs.

## Introduction

In land plants, the sexual structures are developed late in their life-cycle since the gamete-holding organs are initiated on the fully developed mature sporophyte. In contrast, metazoans separate their germline cell lineage very early following gametic fusion. For example, in humans the primordial germ cells developmental fate is established less than a week after fertilization (Richardson and Lehmann, 2010). Flowering plants maintain a population of stem cells that differentiate into various vegetative tissues for the most part of their life but also into the reproductive organs after alteration to the gametophyte phase (reviewed in Berger and Twell, 2011). The specification of maleness and femaleness involves an orchestration of various regulatory mechanisms, and our understanding of such complex gene regulation has improved in recent years (reviewed in Armenta-Medina et al., 2011; Twell, 2011 and Berger and Twell, 2011). Following fusion of gametes through fertilization, plants return to the sporophytic life phase. The alternation between sporophyte and gametophyte generation in the plant's life-cycle implies that genes that are specifically down-regulated or up-regulated in gametes have to be inversely activated or silenced within the non-germline tissues of the dominant phase sporophyte. This silencing and activation require complex and fine-tuned regulatory mechanisms.

Gene regulation contributing to tissue specificity can occur directly through transcription or indirectly by post-translation modification on histones and DNA methylation. Haerizadeh et al. (2006) identified the germline-restrictive silencing factor (GRSF) that specifically represses a sperm cell gene in non-germline cells of lily. However, gene regulation by transcription factors is one type of regulatory mechanism. Gene expression modulation at the transcription level also encompasses epigenetic regulation that leads to changes on the DNA or histone status to block or guide the expression of target genes in the locus vicinity of the changes. Epigenetics is the study of traits, which are defined as “stably heritable phenotypes resulting from changes in a chromosome without alterations to its DNA sequences” (Berger et al., 2009). DNA 5-methyl-cytosine modification (DNA methylation; 5mC) has a repressive nature while histone moieties can have either a positive or a negative effect on gene regulation at the locus where the histone modification occurred. The types of covalent modification on histones vary. Specific acetylated forms, such as H3K9ac, H3K18ac, and H3K27ac or the ubiquitination of H2Bub are epigenetic marks that lead to gene activation, while histone methylation has a less define response on gene expression. As such, the specific methylated amino acid, its hypermethylated state or the surrounding epigenetic context can all lead to a different gene activation/repression status (Liu et al., 2010). In some instance, histone methylation can acts as a repressive mark as it is the case for H3K9me2, H3K27me1, and H3K27me3, or it can be interpreted as a activator mark when found on H3K4me3, H3K9me3, H3K36me2, and H3K36me3. The gene regulation of some histone methylation marks, like H3K4me1 and H3K4me2, is modulated by the other epigenetic marks at that same locus. As such, H3K4me1 and H3K4me2 can act as either a repressive or an activating mark depending on the neighboring epigenetic context. Another type of epigenetic control is emphasized through the use of histone variants, as shown by promoters with a H3.3 variant enrichment that are transcriptionally more active (Shu et al., 2014).

Advances in microarray and DNA-sequencing technologies have allowed the recent expansion of epigenetic modification analyses on many model organisms. Whole-genome tiling array technologies were utilized for not only gene expression profiling including alternative splicing but also the investigation of DNA-methylation (Mockler et al., 2005; Gregory et al., 2008), which have a conserved role in silencing gene expression (Martienssen and Colot, 2001). Microarray technology was also applied to the post-translational modifications of histones (Zhu et al., 2001; Dindot et al., 2009; Moghaddam et al., 2011). Next-generation ultrahigh-throughput sequencing is also actively utilized for exploring the epigenetic modifications of DNA and histone status (Cokus et al., 2008; Ma et al., 2011; Marques et al., 2011). Both methods have investigated the epigenetic characteristics of various tissues of *Arabidopsis thaliana*, making *Arabidopsis* one of the most extensively studied model plant for epigenetic studies.

Recent studies have identified a subset of flowering plant genes that show preferential or enriched expressions in germline cells (Steffen et al., 2007; Borges et al., 2008; Wuest et al., 2010; Drews et al., 2011). These genes are, by definition, suppressed or down regulated in the non-germline tissues. Although many studies have shown the relationships between the tissue-specific gene expressions and the status of epigenetic traits, the mechanisms of epigenetic suppression or down-regulation of germline genes in non-germline tissues are poorly understood. Hence, in this study, we explored the nature of epigenetic marks that are likely associated with long-term gene repression of germline genes in non-germline cells.

## Materials and methods

### Genes with epigenetic marks

Genes with H3K4me1, H3K4me2, and H3K4me3 in 3-week old seedlings were identified from the genomic coordinates of these histone modification marks provided by Zhang et al. (2009). Genes with H3K9me2 (i.e., target genes of H3K9me2) in 3wk-old shoots were selected using the pre-processed sequencing data deposited in Gene Expression Omnibus (GEO) repository in NCBI, GSE12383 (Bernatavichute et al., 2008). Each sequence used in the original paper by Bernatavichute and colleagues was tagged with Z-score of log-ratio between Cy5 (H3K9me2 signal) and Cy3 (H3 signal), and those with Z-score higher than 0.2 were extracted and mapped onto TAIR9 *Arabidopsis* genome sequence using Bowtie (Langmead et al., 2009). Genes overlapping the coordinates of selected probe sequences were regarded as targets of H3K9me2. All the remaining epigenetic marks in a given tissue were extracted directly from the data of corresponding papers listed in Table 1; their respective role is indicated at the end of each epigenetic mark name: “(r),” repression-related; “(a),” activation-related; and “(a/r)” activation or repression depending on other accompanying epigenetic marks.

**Table 1:** **Epigenetic studies using somatic tissues of *Arabidopsis thaliana***.

**Tissue**	**Marks**	**References**
Seedlings (10 days old)	5mC(r), H2Bub(a), H3K27me1(r), H3K27me3(r), H3K36me3(a), H3K4me2(a/r), H3K4me3(a), H3K9me2(r), H3K9me3(a)	Turck et al., 2007; Zhang et al., 2007; Roudier et al., 2011
Seedlings (3 weeks old)	H3K4me1(a/r), H3K4me2(a/r), H3K4me3(a)	Zhang et al., 2009
Seedlings (5 days old, dark-grown)	H3K27ac(a), H3K27me3(r), H3K9ac(a), H3K9me3(a)	Charron et al., 2009
Leaves	H3K27me3(r), H3.3.TTS(a)^*^, H3.3.TTS.Promoter(a)^*^, H3.3.Promoter(a/r)^*^	Lafos et al., 2011; Shu et al., 2014
Shoot apical meristem	H3K27me3(r)	Lafos et al., 2011
Shoots (3 weeks old)	H3K9me2(r)	Bernatavichute et al., 2008
Roots (10 days old)	H3K27me3(r), H3K4me3(a)	Roudier et al., 2011
Aerial tissue (2 weeks old)	H3K4me2(a/r), H3K4me3(a), H3K9Ac(a), H3K9me2(r), H3K18Ac(a), H3K27me1(r), H3K27me3(r), H3K36me2(a), H3K36me3(a), 5mC(r)	Luo et al., 2012

* *Genes with H3.3 mark were grouped by the position of H3.3: H3.3.TTS, H3.3 near transcription termination sites (TTS); H3.3.Promoter, H3.3 in promoter; H3.3.TTS.Promoter, H3.3 near TTS and in promoter*.

### *Arabidopsis* germline genes

Borges et al. (2008) reported 81 *Arabidopsis* genes that are preferentially expressed in sperm cells. For female germline genes, we combined the results from Wuest et al. (2010), Drews et al. (2011), and Steffen et al. (2007) and extracted 855 genes in total that exhibit preferential expression in female gamete tissues. These female genes were further classified into six groups: 165, 157, 125, 16, and 11 genes that are specifically up-regulated in egg cell, synergid cells, central cell, antipodal cells, and embryo sac, respectively, and 381 genes enriched in female gamete tissues as a whole but not in a particular tissue (Table 2, Supplementary Data 1). Proportion of genes carrying specific epigenetic modification was calculated by dividing the number of cell-type genes with an epigenetic modification by the total number of genes of that cell-type. For the calculation of proportion of germline genes with epigenetic marks, germline genes in each group were considered separately (1 group for male and 7 groups for female) or combined by sex (81 male germline genes and 855 female germline genes). Statistically significance of the data was determined by using Pearson coefficient with *t*-test *p*-values.

**Table 2:** **Germline- and gamete-specific genes of *Arabidopsis thaliana***.

	**Male**	**Female**					
	**Sperm**	**Egg**	**Synergid Cell**	**Central Cell**	**Antipodal Cell**	**Embryo Sac**	Female OCC^*^
Genes	81	165	157	125	16	11	381

* *Female OCC, Female Other Cell-type Combination*.

*Male specific genes from Borges et al. (2008); Female specific genes from Wuest et al. (2010); Drews et al. (2011); Steffen et al. (2007)*.

### Small RNAs from TE genes

Small RNA sequencing data were collected from various studies using *Arabidopsis* (Axtell et al., 2006; Kasschau et al., 2007; Montgomery et al., 2008; Fahlgren et al., 2009; Moldovan et al., 2010) and mapped to the TAIR9 *Arabidopsis* genome sequence using Bowtie (Langmead et al., 2009). Sequences that were uniquely aligned within the 30 *Arabidopsis* germline specific transposable elements (TEs) were retained and scored by their length (Supplementary Data 2).

### Gene ontology term enrichment test

Gene ontology (GO) term enrichment analysis was performed by goEAST with default parameters, which included multi-test adjustment using Yekutieli method (Zheng and Wang, 2008).

## Results

### Epigenetic marks on *Arabidopsis* germline genes in different non-germline tissues

#### Epigenetic marks of germline genes in seedlings

**Figure 1:**
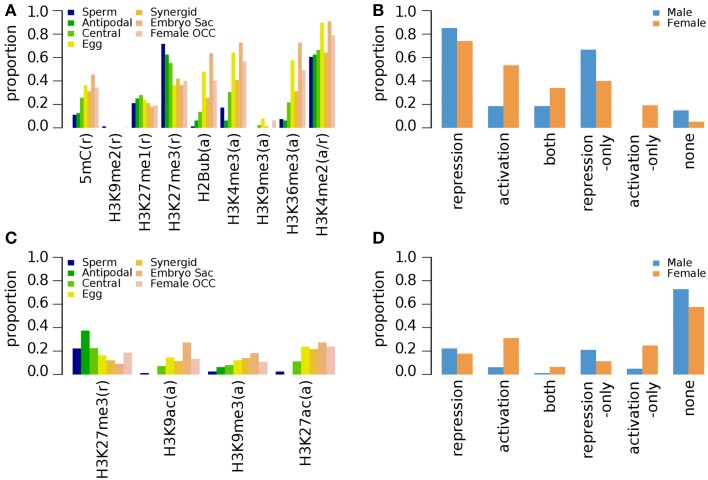
**Distribution of DNA methylation (5mC) and specific histone modifications on *Arabidopsis* germline genes in seedlings**. Genes were selected for their specific expression in one of six different reproductive cell type classes. **(A,B)** Epigenetic marks on germline genes in 10 day-old seedlings grown under normal light conditions. **(C,D)** Epigenetic marks on germline genes in 10 day-old seedlings grown under dark conditions. For **(B,D)**, epigenetic marks are grouped by their repressive or activating nature and the genes are grouped by male and female germline specific expression. Proportion was calculated by dividing the number of cell-type genes with an epigenetic modification by the total number of genes of that cell-type. Female OCC, Female Other Cell-type Combination.

Seedlings are the most extensively examined material for investigating genome-wide epigenetic modifications in *Arabidopsis* (Table 1, epigenetic marks and references therein). Ten days old seedlings (10d-old) were examined for 5mC, H2Bub, H3K27me1, H3K27me3, H3K36me3, H3K4me2, H3K4me3, H3K9me2, and H3K9me3 (Turck et al., 2007; Zhang et al., 2007; Roudier et al., 2011), and seedlings grown under dark condition for 5 days were studied for H3K9me3, H3K27me3, H3K9ac, and H3K27ac (Supplementary Data 3, 4, respectively) (Charron et al., 2009). A complementary study examined H3K4me1, H3K4me2, and H3K4me3 marks in 3 weeks old (3wk-old) seedlings (Supplementary Data 5) (Zhang et al., 2009). Among the repression-related marks found in the non-germline tissues of the 10d-old seedling (grown under normal condition), H3K27me3 was shown to be more abundant at sperm cell-specific genes and those with enriched expression in antipodal cell-specific and central cell-specific genes (Figure 1A). For genes over-expressed in female germlines other than antipodal genes, the proportion of germline genes with DNA methylation (5mC) is comparable between the different female cell type genes (Figure 1A). In contrast, H3K9me2, another repression-related mark, is nearly absent for germline genes, while H3K27me1 (also repression-related) occupies ~20% of germline genes regardless of germline tissues in which their expression is enriched (Figure 1A). Among all epigenetic marks, H3K4me2, which can be related to either activation or repression depending on other accompanying epigenetic marks, is the most common epigenetic mark in 10d-old seedlings for female germline genes. However, H3K27me3 remains as the most common mark for male germline genes (i.e., those that are preferentially expressed in sperm cells) in the same tissues (Figure 1A). When germline genes are grouped by sex-type and compared to activity state, the majority of germline genes have one or more types of repression-related marks in 10d-old seedlings grown under normal conditions, although the fraction of female germline genes with activation-related marks is also high compared to male germline genes (Figure 1B).

In 5 days old (5d-old) dark-grown seedlings, H3K27me3 (*t*-test *p*-value of 0.001) and H3K9me3 (*t*-test *p*-value 0.008) mark substantially less male and female germline genes (Figure 1C) compared to 10d-old seedlings grown in normal condition (Figure 1A). The fractions of germline genes marked by other epigenetic marks are also low in general in 5d-old dark-grown seedlings (Figure 1C), suggesting that the environmental conditions might have influenced the epigenetic modification status. Nevertheless, the fraction of germline genes having activation-related marks is still higher for female germline genes than for male germline genes in 5d-old dark-grown seedling (Figure 1D). Furthermore, in the 5d-old dark-grown seedling, most germline-specific genes have lost most of their regulatory histone marks as shown in Figure 1D.

**Figure 2:**
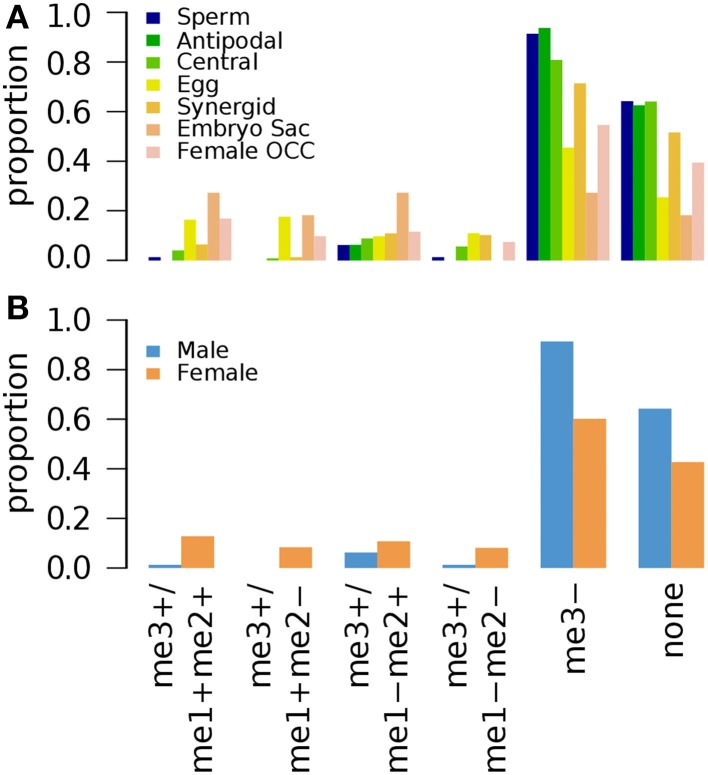
**Distribution of different combinations of H3K4me1, H3K4me2, and H3K4me3 in 3-week old seedlings**. Genes were selected for their specific expression in one of six different reproductive cell type classes. **(A)** Proportion of different combination of H3K4 methylation states in six different reproductive cell type classes. **(B)** Total H3K4 methylation states in male and female genes. Proportion was calculated by dividing the number of cell-type genes with an epigenetic modification by the total number of genes of that cell-type. Female OCC, Female Other Cell-type Combination.

*Arabidopsis* seedlings grown for 3 weeks (3wk) under normal conditions were examined for different combinations of epigenetic marks H3K4me1, H3K4me2, and H3K4me3 (Zhang et al., 2009). A small fraction of germline genes has these marks regardless of the combinations (Figure 2). Zhang et al. (2009) showed that the presence of H3K4me3 for a gene is associated with medium to high level of expression regardless of the type of other accompanying marks (none, either or both of H3K4me1 and H3K4me2). Thus, it is expected to have a small fraction of germline genes to be marked by H3K4me3 with or without H3K4me1 and/or H3K4me2 (Figure 2). We aforementioned that activation-related marks are more common for female germline genes in 5d/10d-old seedlings regardless of growth condition (Figure 1). Likewise, a larger fraction of female germline genes are marked by H3K4me3 with either or both of H3K4me1 and H3K4me2 (me3+/me1+me2+, me3+/me1+me2–, and me3+/me1–me2+) or H3K4me3 alone (me3+/me1–me2–) compared to male germline genes (Figure 2), although these proportions are generally small. Sperm genes show a depletion of the H3K4me3 marks, where around 90% of sperm genes show an absence of this specific activator mark and around 60% of sperm genes are without any H3K4 methylation moieties (Figure 2). This was also observed at antipodal cell and central cell genes, where those female cell type genes follow sperm cell genes regulation between 10d-old seedlings and 3wk-old seedlings (Figures 1A,B, 2, respectively).

#### Epigenetic marks on germline genes in aerial tissue

**Figure 3:**
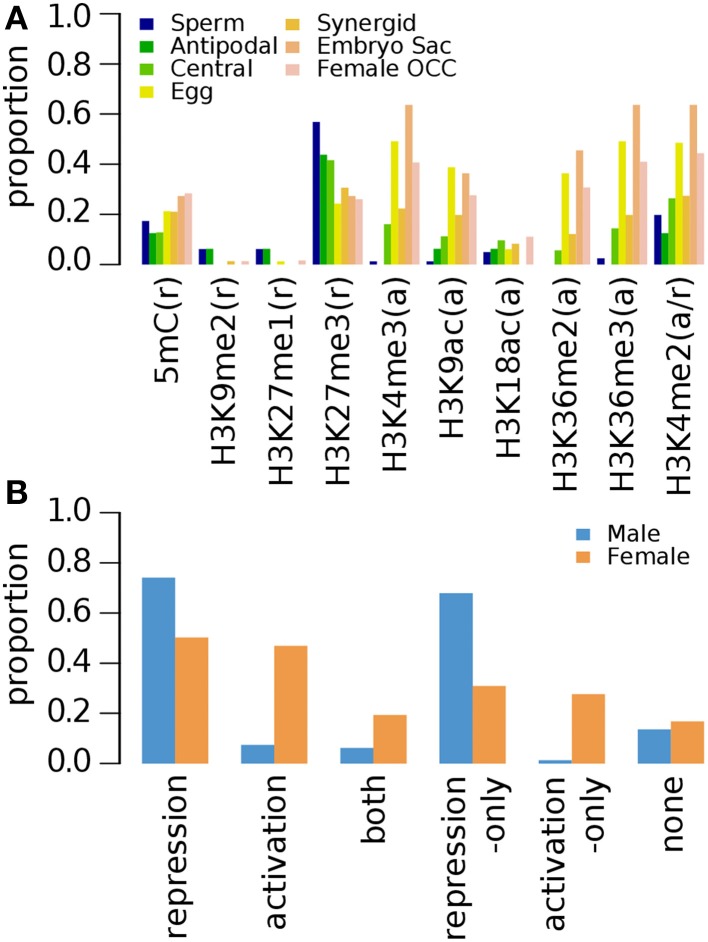
**Distribution of DNA methylation (5mC) and specific histone modifications on *Arabidopsis* germline genes in 2 week-old (2wk-old) aerial tissues**. Genes were selected for their specific expression in one of six different reproductive cell type classes. **(A)** Proportion of germline genes with specific epigenetic marks in aerial tissues. **(B)** Epigenetic marks grouped by their repressive or activating nature and the genes grouped by male and female germline specific expression. Proportion was calculated by dividing the number of cell-type genes with an epigenetic modification by the total number of genes of that cell-type. Female OCC, Female Other Cell-type Combination.

A recent investigation of nine histone modification marks and DNA methylation on 2 week old (2wk-old) aerial tissue was conducted by Luo et al. (2012) (Supplementary Data 6). H3K27me3 is the most common mark among the repression-related marks for germline genes except for those with enriched expression in the female gamete as a whole (Figure 3A). Large proportion of female germline genes also has activation-related marks, some of which are marked by only activation-related marks, whereas only a small fraction of male germline genes, have activation-related marks (Figure 3B), which results in the similar overall distribution of epigenetic marks as in that of 10d-old seedlings when marks are grouped by the regulatory effects and the genes were grouped by sex (Figure 1B compare to Figure 3B): Pearson's correlation coefficients: 0.98 for male (*p*-value 0.0006) and 0.89 for female (*p*-value 0.0168). The 2wk-old seedling dataset shows a relatively weak abundance of activating acetylated histone marks in all genes, but again, the depletion is more pronounced in sperm cell specific genes (Figure 3).

#### Epigenetic marks on germline genes in roots, shoots, leaves and shoot apical meristem

**Figure 4:**
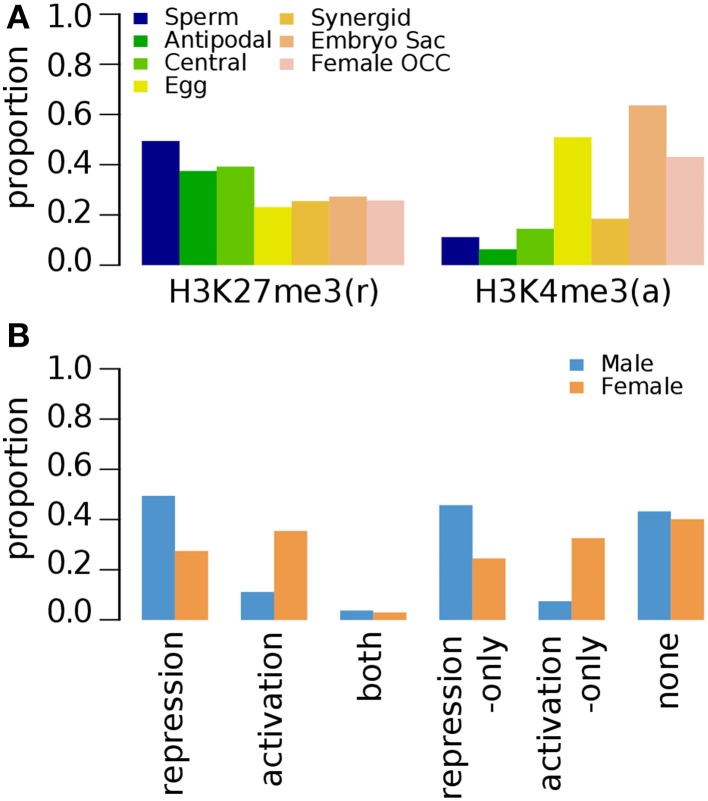
**Distribution of specific histone modifications (H3K27me3 and H3K4me3) on *Arabidopsis* germline genes in root tissues**. Genes were selected for their specific expression in one of six different reproductive cell type classes. **(A)** Genes with H3K27me3 and H3K4me3 in six different reproductive cell type classes. **(B)** Epigenetic marks grouped by their repressive or activating nature and the genes grouped by male and female germline specific expression. Proportion was calculated by dividing the number of cell-type genes with an epigenetic modification by the total number of genes of that cell-type. Female OCC, Female Other Cell-type Combination.

Epigenetic studies on other tissues are limited to a few marks, such as H3K27me3, H3K9me2, and H3K4me3 (Table 1) (Supplementary Data 7–10). In roots, H3K27me3 (repression-related) is associated with ~50% of sperm-preferential genes and below ~40% of female germline genes regardless of their preferred female germline tissue (Figure 4A). The occupancy of H3K27me3 in female germline genes in roots further decreases when all female germline genes are combined (Figure 4B). The activation-related mark H3K4me3 occupies less than 20% of germline genes specifically over-expressed in sperm, antipodal, central and synergid cells. However, a relatively high proportion of female germline genes specific to other germline tissues appear to have elevated the overall occupancy of H3K4me3 in female germline genes (Figure 4).

**Figure 5:**
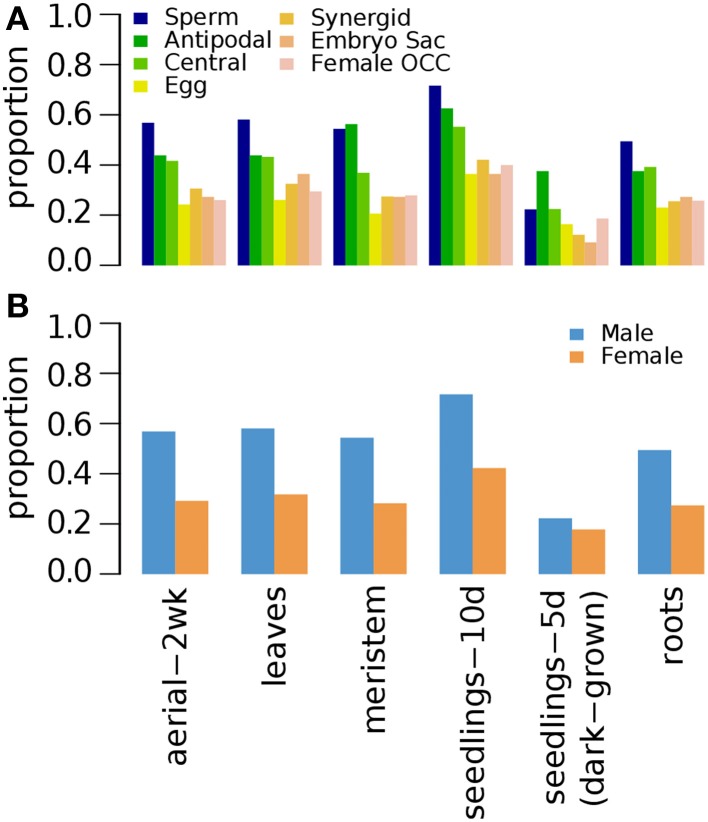
**Distribution of H3K27me3 histone modification on *Arabidopsis* germline genes in various non-germline tissues**. Genes were selected for their specific expression in one of six different reproductive cell type classes. **(A)** Proportion of germline genes marked by H3K27me3 in six different non-germline tissues. **(B)** Male and female germline specific genes marked by H3K27me3 in six different non-germline tissues. Proportion was calculated by dividing the number of cell-type genes with an epigenetic modification by the total number of genes of that cell-type. Female OCC, Female Other Cell-type Combination; Aerial-2wk, 2 week-old aerial tissues; meristem, shoot apical meristem; seedlings-10d, 10 day-old seedlings; seedlings-5d (dark-grown), 5 day-old seedlings grown in dark condition.

H3K27me3 modification (Supplementary Data 11) was similarly distributed in seedlings (10d-old), roots, shoot apical meristem and leaves, with 10d-old seedlings having the most abundant with nearly 80% occupancy (Figure 5). However, light-deprived seedlings have fewer germline genes marked by H3K27me3 (Figure 5) as previously depicted in Figures 1C,D.

**Figure 6:**
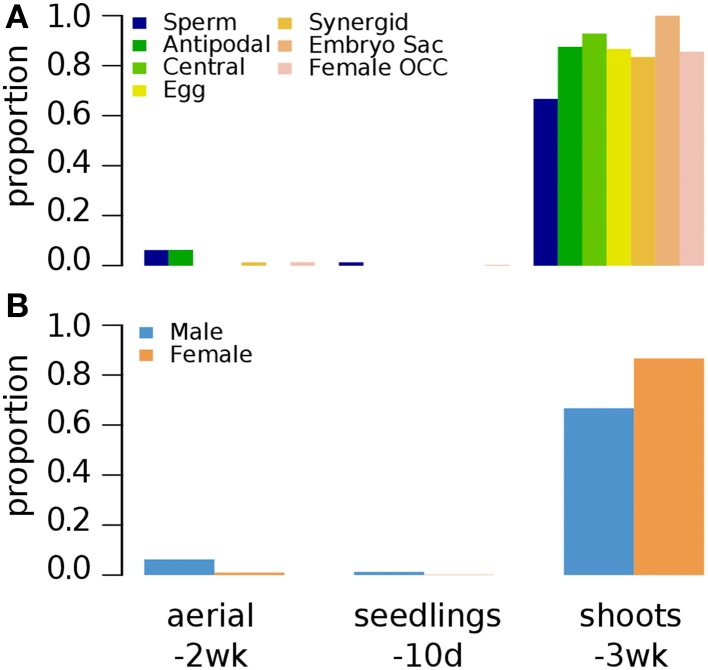
**Distribution of H3K9me2 histone modification on *Arabidopsis* germline genes in various non-germline tissues**. Genes were selected for their specific expression in one of six different reproductive cell type classes. **(A)** Proportion of germline genes marked by H3K9me2 in three different non-germline tissues. **(B)** Proportion of male and female genes marked by H3K9me2 in three different non-germline tissues. Proportion was calculated by dividing the number of cell-type genes with an epigenetic modification by the total number of genes of that cell-type. Female OCC, Female Other Cell-type Combination; Aerial-2wk, 2 week-old aerial tissues; seedlings-10d, 10 day-old seedlings; shoots-3wk, 3 week-old shoots.

Three week old (3wk-old) shoots were examined for H3K9me2, a repression-related mark (Table 1) (Bernatavichute et al., 2008). In contrast to 10d-old and 2wk-old aerial tissues where H3K9me2 is nearly absent for germline genes, majority of germline genes are marked by H3K9me2 in 3wk-old shoots, suggesting that H3K9me2 plays an important role for the down-regulation of germline genes in shoots (Figure 6).

#### Epigenetic marks on germline genes in non-germline tissues as a whole

**Figure 7:**
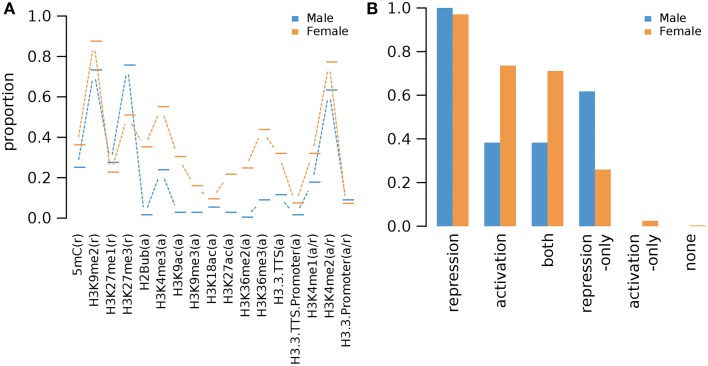
**Proportions of *Arabidopsis* germline genes having epigenetic marks in one or more of non-germline tissues**. **(A)** Individual epigenetic marks. **(B)** Epigenetic marks grouped by their repressive or activating nature and the genes grouped by male and female germline specific expression. Proportion was calculated by dividing the number of cell-type genes with an epigenetic modification by the total number of genes of that cell-type. Female OCC, Female Other Cell-type Combination.

We combined the epigenetic mark information for germline genes in all examined non-germline tissues (Supplementary Data 12). The combined analysis of epigenetic mark data in various non-germline tissues shows that different epigenetic marks are found in different fraction of male and female germline genes in non-germline tissues as a whole but the overall trends are similar: Pearson's correlation coefficient 0.80 (*p*-value: 9.7e-5) (Figure 7A). As expected, all male germline genes and almost all female germline genes (97%, i.e., 830 genes) have one or more repression-related epigenetic marks in non-germline tissues (Figure 7B). Among the repression-related marks, H3K9me2 marks the majority of all female germline genes in one or more of non-germline tissues (Figure 7A and Supplementary Figure S1). For male germline genes, H3K27me3 is the most common repression-related mark in non-germline tissues (75%) and H3K9me2 also marks the majority of sperm-preferential genes (73%) (Figure 7A). Similarly, H3K27me3 is the second most common repression-related mark on female germline genes in non-germline tissues (51%) (Figure 7A). Interestingly, a substantial fraction of female germline genes (74%) have one or more types of activation-related marks in non-germline tissues, and 21 female germline genes have only activation-related marks in non-germline tissues (Figure 7B). In comparison, the fraction of male germline genes with activation-related marks in non-germline tissue is much lower than that of female germline genes, whereas no male germline-genes is marked only by activation-related marks (Figure 7B). The variant H3.3, known to be linked to up-regulated promoters (Shu et al., 2014), was found to have very low abundance (less than 10%) of all germline-specific genes in non-germline tissues (Figure 7A).

### Germline genes with only repression-related marks in non-germline tissues

As germline genes are found to be up-regulated only in germline tissues, a substantial fraction of the germline genes are associated with only repression-related marks: 50 out of 81 male germline genes (62%) and 222 out of 855 female germline genes (26%). GO term enrichment analysis performed by goEAST (Zheng and Wang, 2008) reveals that nine GO terms are enriched in 50 male germline genes that have only repression-related marks in non-germline tissues, which are biological processes related to gametophyte development or reproduction (Table 3). However, no particular GO term is enriched in 222 female germline genes that have only repression-related marks.

**Table 3:** **GO term enrichment analysis of male germline genes with repression-only marks in non-germline tissues**.

**GOID**	**Term**	**Gene symbols**	FDR^*^
GO:0048235	Pollen sperm cell differentiation	MGH3; DAZ1; HAP2; DAZ3	3.94E-05
GO:0048232	Male gamete generation	MGH3; DAZ1; HAP2; DAZ3	6.52E-05
GO:0055046	Microgametogenesis	MGH3; DAZ1; HAP2; DAZ3	7.07E-05
GO:0019953	Sexual reproduction	MGH3; DAZ1; HAP2; DAZ3; KPL	0.00165
GO:0009555	Pollen development	MGH3; DAZ1; HAP2; DAZ3; KPL	0.00613
GO:0022412	Cellular process involved in reproduction in multicellular organism	MGH3; DAZ1; HAP2; DAZ3	0.007707
GO:0007276	Gamete generation	MGH3; DAZ1; HAP2; DAZ3	0.010165
GO:0044702	Single organism reproductive process	MGH3; DAZ1; HAP2; DAZ3; KPL	0.010412
GO:0048229	Gametophyte development	MGH3; DAZ1; HAP2; DAZ3; KPL	0.036192

* *False Discovery Rate after multiple-test adjustment by Yekutieli method (Zheng and Wang, 2008)*.

**Figure 8:**
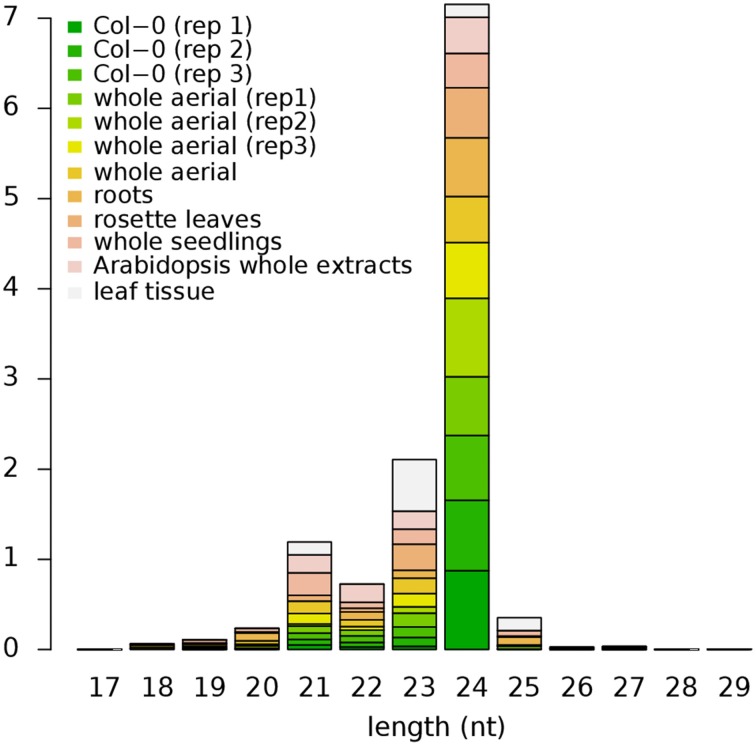
**Size distribution of small RNAs mapped within *Arabidopsis* TEs**. Small RNAs of 24-nt long in length are the most abundant class in all datasets except for the leaf tissue where 23-nt long small RNAs are more abundant. Height of each block represents their respective proportion. Col-0, Colombia; rep1-3, replicate 1-3, respectively; TEs, Transposable Elements.

Among the male and female germline genes, 30 are annotated as TEs, 29 of which have one or more types of repression-related epigenetic marks (Supplementary Data 13). While DNA methylation is associated with less than 40% of all germline genes (Figure 7A), 27 germline TEs (90%) are methylated in non-germline tissues. This is consistent with the previous studies that showed the silencing of retrotransposon DNA in plants through DNA methylation (Hirochika et al., 2000; Miura et al., 2001). Along with DNA methylation, we also observed small RNAs being derived from these germline TEs in non-germline tissues. The most abundant class of small RNAs is 24-nt long small interfering RNAs (siRNAs), which is known to be over-expressed in and around transposons and retroelements in *Arabidopsis* along with 23-nt long siRNAs (Kasschau et al., 2007) (Figure 8). The observation of germline TEs having both DNA methylation and 24-nt long siRNAs in non-germline tissues is in agreement with the reported involvement of siRNAs in the gene silencing pathway via RNA-directed DNA methylation (Hamilton et al., 2002).

### Germline genes with only activation-related marks in non-germline tissues

While the repression-related epigenetic marks and small ncRNAs are likely involved in down-regulation of germline genes in non-germline tissues in an epigenetic manner, 21 germline genes are associated with only activation-related marks (Table 4). The most common activation-related mark is H3K4me3 (16 genes). Interestingly, in 10d-old seedlings these 16 genes are co-marked by H3K4me2, which acts as either activation- or repression-related mark- depending on accompanied marks. Similarly, among 11 genes that have H3K4me3 in 2wk-old aerial tissue, 9 also have H3K4me2 marks.

**Table 4:** **Description of protein function of germline-specific genes with activation epigenetic marks only**.

	**Activation-related marks**	**Description**
AT2G42930	H2Bub; H3K36me3	Glycosyl hydrolase family protein 17
AT1G10330	H3K36me3; H3K4me3; H3K27ac	Pentatricopeptide (PPR) repeat-containing protein
AT2G35730	H3.3.TTS	Heavy-metal-associated domain-containing protein
AT3G58100	H3K36me3; H3K4me3; H3K9ac; H3K27ac	PDCB5 (PLASMODESMATA CALLOSE-BINDING PROTEIN 5);
AT3G62320	H2Bub	Nucleic acid binding
AT4G16440	H3K36me2; H3K36me3; H3K4me3; H3K9ac; H3.3.TTS; H2Bub; H3K9me3; H3K27ac	Ferredoxin hydrogenase
AT5G09240	H3K9ac; H3.3.TTS; H3K36me3; H3K4me3; H3K27ac	Transcriptional coactivator p15 (PC4) family protein
AT5G60270	H3K36me3; H3K4me3; H3.3.TTS; H2Bub	Lectin protein kinase family protein
AT3G04410	H3.3.TTS.Promoter; H3K4me3; H3K27ac	Transcription factor
AT3G09310	H3K4me3; H3K9ac; H3.3.TTS; H2Bub; H3K36me3	Unknown protein
AT3G13682	H3K18ac; H3K36me2; H3K36me3; H3K4me3; H3K9ac; H3.3.TTS; H2Bub; H3K27ac; H3K9me3	LDL2 (LSD1-LIKE2); amine oxidase/electron carrier/oxidoreductase
AT5G03160	H3K36me2; H3K36me3; H3K4me3; H3.3.TTS; H2Bub; H3K27ac	*Arabidopsis* homolog of ATP58IPK
AT1G48780	H3K36me2; H3.3.TTS; H2Bub; H3K36me3; H3K4me3	Unknown protein
AT1G49150	H3K4me3	Unknown protein
AT1G61450	H3K36me3	Unknown protein
AT2G18650	H3K4me3; H3K9ac; H3K36me3	MEE16 (maternal effect embryo arrest 16); protein binding/zinc ion binding
AT3G18120	H3K4me3	F-box family protein-related
AT3G22670	H3K36me3; H3K4me3; H3K9ac; H2Bub; H3K27ac	Pentatricopeptide (PPR) repeat-containing protein
AT4G25560	H3.3.TTS; H2Bub	AtMYB18 (myb domain protein 18); DNA-binding/transcription factor
AT5G06410	H3K18ac; H3K36me3; H3K4me3; H3K9ac; H2Bub	DNAJ heat shock N-terminal domain-containing protein
AT5G15760	H3K4me3; H3K9ac; H2Bub; H3K36me3	Plastid-specific 30S ribosomal protein 3, putative/PSRP-3, putative

## Discussion

Current published plant genomic studies cover only a fraction of the total known epigenetic marks (Tessarz and Kouzarides, 2014) while some epigenetic marks with known regulatory function have not been examined at a genome-wide level yet. Here we report the current state of knowledge regarding epigenetic regulation of germ-line specific genes. By analyzing a broad range of whole-genome studies, we uncover a spatial and temporal understanding of the epigenetic repression of these genes in the somatic tissues (Table 2).

### Disparity and similarity between male and female regulation

We show that the epigenetic regulation differs between the two types of gamete-specific genes. There is a preponderance of H3K27me3 deposition in male germline-genes while there is a combination of different marks for the female counterpart with a preference for H3K9me2 (Supplementary Figure S1). The molecular machinery involved in the pre- and post-deposition of epigenetic marks could differ between the male- and female-specific expressed genes. As such, different SET domain-containing proteins, methyltransferases, are likely to be involved as well as the recognition machinery that delivers the specific SET proteins to the to-be-repressed loci. The methylation is then followed by recognition from different proteins acting as reader and /or effectors. Different protein recognizes different histone marks leading to a set downstream effect (reviewed in Liu et al., 2010). Although the resulting function of those marks are to shut down specific germline expression in non-germline tissue, the mechanisms used between male and female genes will most likely differ, as different epigenetic marks are accumulated at their respective loci. Another striking difference between the two types of germline gene regulation is the use of the activator marks for the female gametophyte gene regulation (Figures 1–4, 7). Although H3K4me1/2 marks are context dependent, H3K4me3 is a well-established activator mark. As one of the most important lineage cell, the egg cell shows the most of this specific mark (Supplementary Figure S1). Wuest et al. (2010) described a very specific and tight regulation of the *Arabidopsis* egg cell specific genes. As such it is surprising to see an abundance of H3K4me3 at these loci in tissue where their expression is repressed. Although H3K4me3 was rarely seen alone at these loci (Figure 2), hinting to the possibility that H3K4me3 might acts in similar ways as its contextual undermethylated counterparts. In fact, we found that the variant H3.3 was absent on these germline genes (Figure 7A) indicating that H3K4 might act as repressor mark on those loci. The higher ratio of the variant H3.3 to H3 is an indicative of gene activity (Shu et al., 2014). This trend is supported by our current findings. We also highlight a similarity in epigenetic control between the sperm cell-specific genes and some of the female gametophyte cell specific genes. The antipodal cells, the central cell, and the sperm cell gene loci behave in a related pattern (Supplementary Figure S1). This could be the ancestral germline-specific repression mechanism and where the egg cell would have evolved additional regulation mechanisms over time. It would be interesting to test this hypothesis if epigenetic whole-genome analysis were available in the lower plant species like *Marchantia polymorpha* and *Physcomitrella patens*. However, we show here that the repression of gamete-specific genes in the somatic tissue could be encompassed by epigenetic regulation, which could restrict these genes to their expression zones. The mechanism involved to achieve this seems to have recruited different repressive strategies between the two types of germline-specific genes; a predominant H3K27me3 pathway for sperm cell-specific genes and a combination of both activating and repressive marks for the egg cell-specific genes.

### H3K27me3 as a sperm cell-specific gene repression mechanism

In *Arabidopsis*, the mature pollen grain is composed of three cells: a vegetative cell that produces the pollen tube and two sperm cells that are transported down the pollen tube to participate in the double fertilization in the female gametophyte. In this instance, both sperm cells are considered the male germline cells. In somatic tissues, our analysis demonstrated that repressive epigenetic marks were found at the 81 sperm cells specific loci (Figures 1, 3–7). Detection of methylation on lysine K4 and K36 of histone H3 was minimal at those loci in somatic tissues, although the contextual epigenetic mark H3K4me2 was found at a higher level in some tissues (Figures 1, 3). As a context dependent epigenetic mark, H3K4me2 could either act as an activating or a repressive mark. At the vicinity of the 81 sperm cell specific loci, we are tempted to conclude that H3K4me2 could depict a repressive nature only. This is supported in Figure 2, where the diverse composition of methylated H3K4 showed that the presence of the dimethylated status was always low in combination of the other two methylation states known as activator marks. Sperm cell-specific repressor inside the somatic cells is most likely the trimethylated form on H3K27. This histone modification was the most abundant repressive mark found at male germline genes in 10d-old seedlings, 2wk-old seedlings and root tissues (Figures 1, 3, 4, respectively). This finding is agreement with Hoffmann and Palmgren (2013) study using whole male gametophyte. In their study, both K27me3 and K4me2 were detected at high level in pollen-specific loci of the non-pollen tissues. It would be interesting to see if there is a distinction between the nature of the repressive marks between sperm cell specific loci and vegetative cell specific loci found in the somatic cells. Immunofluorescence analyses of global histone methylation marks showed differential methylation states between the generative and the vegetative cell nuclei of the mature bicellular pollen in *Lilium longiflorum* (Okada et al., 2006; Sano and Tanaka, 2010). Trimethylation at K27 was shown to be abundant in most mature non-germline cells of the anthers (O'Brien et al., 2014) including the vegetative cell of the pollen (Sano and Tanaka, 2010; O'Brien et al., 2014) while HK4me2 was abundant only in the vegetative cell (Okada et al., 2006). As such, sperm cell-specific genes in non-germline cells could see their expression repressed through H3K27me3-mediated recruitment of repressor complexes.

### Repression through both activating and repressing marks in female germline-specific genes

The embryo sac of *Arabidopsis* is composed of 7 cells and 8 nuclei: the egg cell and the bi-nucleate central cells that give rise to the embryo and the endosperm respectively, while antipodal cells and synergids do not contribute to the genetic lineage of the offspring but are still components of the female gametophyte. Although H3K9me2 was the most abundant repressive mark, we found a combination of both repressive marks as well as activation marks at the loci of the different female gametophyte specific genes (Supplementary Figure S1). In somatic tissues of male germline-specific genes, repressive marks are dominant (our study and Hoffmann and Palmgren, 2013). It is quite intriguing, that female germline specific genes have a different regulation mechanism where activating marks are present as well as repressive marks. Overall, there is a large variation in epigenetic marks found at the different tissue specific loci of the egg cell, the central cell, and the synergid cell, where repressor marks are more abundant in the central cell followed by synergid cells and in less frequent in the egg cell, while activator marks show an opposite pattern (Figure 1). Regulation of female gametophyte-specific gene expression seems to follow divergent epigenetic pathway depending on the cell type inside the embryo sac. What keeps the egg cell-specific genes repressed in the sporophytic tissue while a large proportion of these genes are host to activator marks is not clear? It is quite possible that the overall repertoire of repressive epigenetic marks is not fully revealed for the egg cell-specific genes and additional coverage with other epigenetic marks are needed. As such, a variety of known repressive marks have not been investigated at the whole genome level to date. Histone methylation on arginine is one of them. Alternatively, recent whole-genome studies of repressive context like the interactive heterochromatic islands (Feng et al., 2014) and the heterochromatic histone variant H2A.W (Yelagandula et al., 2014) have not been integrated in the current studies. Data from these two studies could possibly contribute toward explaining why egg cell-specific genes harbor a large proportion of activator epigenetic marks as compared to repressive marks. Are uncharacterized epigenetic marks able to influence negatively the expression of genes even in the presence of activator marks at their loci? Or alternatively, can activator marks expression potential be made ineffective by either a large abundance of different repressive marks? Can some specific epigenetic mark readers have a higher affinity toward some repressive marks that some activator marks readers toward activator marks? At the molecular level, a mechanism must be in place to shut down the gene expression of female-specific expressed genes in the somatic tissues in spite of the presence of activator marks found at those loci in non-germline tissues.

### Differences in spatial and temporal regulation of the same germline genes

Interestingly, while H3K9me2 is associated with the majority of germline genes in 3wk-old shoots (Figure 6), it is nearly absent from gamete-expressed genes in 10d-old seedlings (Figures 1, 6) and of 2wk-old shoots (Figure 3). This is also the case for the H3K4me2 mark that show dynamic changes, where 10d-old seedlings and 2wk-old shoots have abundance of the mark while 3wk-old shoots show a lack of H3K4 marks altogether (Figure 2). This is not surprising as 10d-old shoot and 2wk-old shoots are developmentally similar. This finding implies that different tissues as well as broader developmental stages use different types of epigenetic marks to repress the same set of genes implying that epigenetic marks are dynamic, versatile, and the type of mark as such is not as important as its inherent property to keep germline genes repressed in the non-gametophytic tissues. The inflorescence and the cauline leaves tissue could contribute toward differences seen between the two tissues in 1 week time frame (2wk-old *vs*. 3wk-old), pointing toward an intriguing regulatory system involving two different gene repression mechanisms.

### Germline-specific TEs regulation in non-germline tissues

In this study we report that 93% of germline specifically expressed TEs are methylated at their respective loci in the non-germline tissues. Germline-specifically expressed TEs raise the question of why such elements are active in these cells. TE transcripts have also been previously reported in rice pollen and germ cells (Russell et al., 2012, 2014). TE genomic DNA methylation occurs through RNA-directed DNA methylation (RdDM) where small interfering RNA (siRNA) directs *de novo* DNA methylation to its cognate homologous DNA region. The DNA-dependent RNA polymerase (RNAP) enzymes IV and V are involved in two different pathways for DNA methylation. The RNAP IV and V complexes are highly similar except for their corresponding largest subunits NRPD1 (At1g63020) and NRPE1 (At2g40030) respectively. RNAP IV acts upstream of RdDM and in conjunction with other protein partners, generates 24 nucleotide long siRNAs, while RNAP V acts downstream of RNAP IV and facilitates *de novo* DNA methylation through siRNA-charged ARGONAUTE 4 (AGO4) at specific targeted loci (reviewed in Haag and Pikaard, 2011). From the 29 germline-expressed TEs, we could identify an enrichment of corresponding 24 nucleotide long siRNAs in somatic cells (Figure 8), revealing that RdRM pathways are involved in TEs repression in non-germline cells. ATGene Express reports that RNA expression of both NRPD1 and NRPE1 are at its lowest in the pollen, which could explain the activation of TEs expression in germline cells while the siRNA would keep the germline-expressed TEs in check in somatic tissue. These results indicate that plants might have evolved a mechanism to specifically and voluntary regulate TEs in their germline. This could in term allow for random selective opportunities through genomic shuffling of the gamete genetic material, a mean to adaptive selection.

## Concluding remarks

We used whole genome analysis to show that a large proportion of germline-specific genes show repressive epigenetic marks at their respective loci in somatic tissues. Repressive marks, H3K9me2 and H3K27me3 could be used at these loci to maintain the status of the germline-specific genes in a repressed state outside the germline cells. These two marks (H3K9me2 and H3K27me3) were found to be abundant at sperm cell-specific genes making H3K9me2 and H4K27me3 the key epigenetic modifications behind the repressed states of the genes in the non-germline cells. A similar situation was also observed in the case of egg cell-specific genes. However, a larger abundance of activator marks were also present at female germ-line cells gene loci. Thus, our study shows that epigenetic control of gene expression is likely to be a dominant mechanism for repressing germline genes in somatic tissues, paving the way for discovering additional marks in future large-scale genomic studies.

## Conflict of interest statement

The authors declare that the research was conducted in the absence of any commercial or financial relationships that could be construed as a potential conflict of interest.

## Supplementary material

Supplementary Data 1 **List of genes specifically expressed in different germline cells**.

Supplementary Data 2 **Normalized read-counts of small RNA sequences (count-per-million) mapped within germline transposable elements**.

Supplementary Data 3 **Presence of various epigenetic marks on germline genes in 10 day-old seedlings**.

Supplementary Data 4 **Presence of various epigenetic marks on germline genes in 5 day-old dark-grown seedlings**.

Supplementary Data 5 **Presence of various epigenetic marks on germline genes in 3 week-old seedlings**.

Supplementary Data 6 **Presence of various epigenetic marks on germline genes in 2 week-old aerial tissues**.

Supplementary Data 7 **Presence of various epigenetic marks on germline genes in roots**.

Supplementary Data 8 **Presence of various epigenetic marks on germline genes in 3 week-old shoots**.

Supplementary Data 9 **Presence of various epigenetic marks on germline genes in leaves**.

Supplementary Data 10 **Presence of various epigenetic marks on germline genes in shoot apical meristem**.

Supplementary Data 11 **Presence of the epigenetic mark, H3K27me3 on germline genes in 2 week-old aerial tissues, leaves, shoot apical meristem, 10 day-old seedlings, 5 day-old dark-grown seedlings and roots**.

Supplementary Data 12 **Combined counts and proportions of germline genes with various epigenetic marks in non-germline tissues**.

Supplementary Data 13 **Presence of various epigenetic marks on germline TEs in non-germline tissues**.

Supplementary Figure S1 **Proportions of germline genes with various epigenetic marks in non-germline tissues**. Related germline tissues are in X-axis, and the epigenetic marks are in Y-axis. Proportions are indicated in cells and are color-coded as shown in the legend at top-right side of the figure. Proportion: Number of genes carrying specific epigenetic modification was calculated by dividing the amount of cell-type genes with an epigenetic modification by the total amount of genes of that cell-type.
