# Supplementary figures and images for: Epigenetic landscape of germline specific genes in the sporophyte cells of Arabidopsis thaliana
Source: Front Plant Sci. 2015 May 13;6:328. doi: 10.3389/fpls.2015.00328 (PMC4429549; doi:10.3389/fpls.2015.00328)

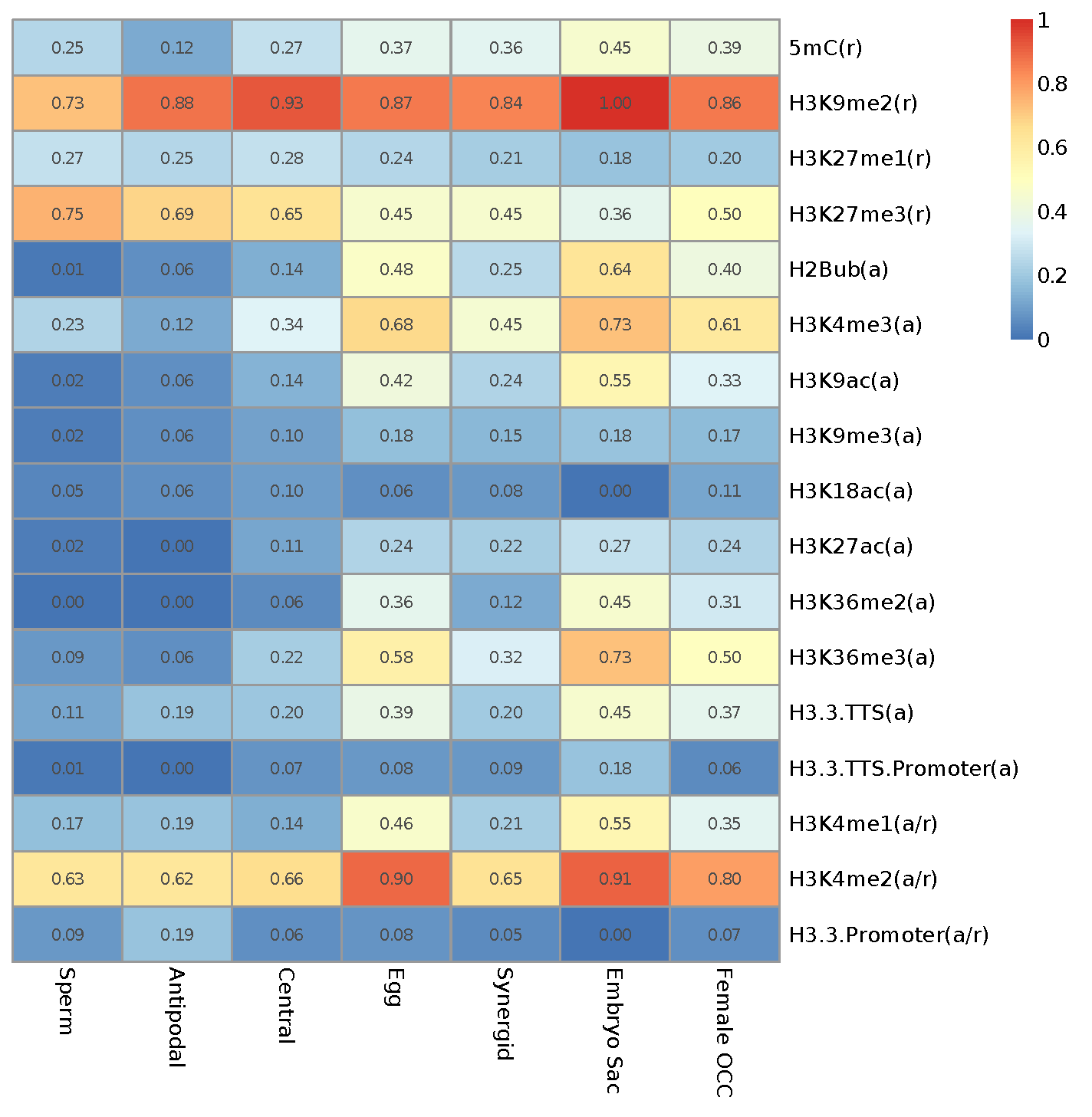

Supplement: Supplementary Figure S1 — Proportions of germline genes with various epigenetic marks in non-germline tissues. Related germline tissues are in X-axis, and the epigenetic marks are in Y-axis. Proportions are indicated in cells and are color-coded as shown in the legend at top-right side of the figure. Proportion: Number of genes carrying specific epigenetic modification was calculated by dividing the amount of cell-type genes with an epigenetic modification by the total amount of genes of that cell-type. [file Image1.TIF]
